# The survival, development, and reproduction of *Gonipterus platensis* (Coleoptera: Curculionidae) on the main *Eucalyptus* (Myrtaceae) genotypes planted in Brazil

**DOI:** 10.7717/peerj.13698

**Published:** 2022-08-01

**Authors:** Nádia Cristina de Oliveira, Murilo Fonseca Ribeiro, Angelo Ottati, Wagner de Souza Tavares, José Eduardo Serrão, José Cola Zanuncio, Ronald Zanetti, Carlos Frederico Wilcken

**Affiliations:** 1 Departamento de Proteção Vegetal, Faculdade de Ciências Agronômicas, Universidade Estadual Paulista (UNESP), Botucatu, São Paulo, Brazil; 2 Superintendência Federal de Agricultura, Pecuária e Abastecimento no Estado do Maranhão, Ministério da Agricultura, Pecuária e Abastecimento, São Luís do Maranhão, Maranhão, Brazil; 3 Riau Andalan Pulp and Paper, Asia Pacific Resources International Holdings Ltd, Pangkalan Kerinci, Riau, Indonesia; 4 Departamento de Biologia Geral, Universidade Federal de Viçosa, Viçosa, Minas Gerais, Brazil; 5 Departamento de Entomologia/BIOAGRO, Universidade Federal de Viçosa, Viçosa, Minas Gerais, Brazil; 6 Departamento de Entomologia, Universidade Federal de Lavras, Lavras, Minas Gerais, Brasil

**Keywords:** Bioecology, *Eucalyptus camaldulensis*, *Eucalyptus grandis*, Eucalypt hybrid, Eucalyptus snout beetle, *Eucalyptus urophylla*

## Abstract

**Background:**

*Gonipterus platensis* Marelli (Coleoptera: Curculionidae) is the main defoliating beetle of *Eucalyptus* L’Hér. (Myrtaceae) plants worldwide. The suitability of *Eucalyptus* to this pest varies among host plant genotypes. The objective of this study was to evaluate the development, reproduction, and survival of *G. platensis* on *Eucalyptus* species and hybrids to assess their suitability to this insect pest in Brazil.

**Methods:**

The survival, development, and reproduction parameters were evaluated with *G. platensis* feeding leaves of *Eucalyptus camaldulensis* Dehnh.,* Eucalyptus grandis* W. Hill.,* Eucalyptus urophylla* S.T. Blake and on the hybrids of *E. grandis* ×*E. urophylla* ‘H13’ and ‘VR3748’ in the laboratory.

**Results:**

The duration of the larval stage of *G. platensis* was shorter on *E. urophylla.* The pupal stage and the period from larva to adult were equally shorter on *E. urophylla* and *E. camaldulensis*. The viability of instars of this insect was low on both *E. grandis* and *E. camaldulensis*. The complete lifespan, oviposition period and reproduction parameters of *G. platensis* were greater on *E. urophylla*, lower on *E. camaldulensis* and *E. grandis*, and intermediate on both hybrids tested.

**Synthesis:**

*Eucalyptus urophylla* is the most suitable host for *G. platensis* survival, development, and reproduction, while *E. grandis* and *E. camaldulensis* are the least suitable.

## Introduction

Planted forests cover around 131 million hectares in the world (FAO, 2020). Brazil is one of the biggest producers with 9.55 million hectares in 2021 to produce raw material for bioenergy, firewood, laminate, pulp and paper, timber, and wall panels (Ribeiro, Rodrigues & Ballarin, 2020; IBÁ, 2021). *Eucalyptus* L’Hér. (Myrtales: Myrtaceae) is the most prominent plant genus in Brazilian forest plantations with around 78% of the area planted (IBÁ, 2021). The rapid growth, easy regeneration and cultivation, adaptation to different geographic location places, multiple uses, among others, contribute to the expansion of *Eucalyptus* in the world (Tomé et al., 2021). The large areas with forest plantations and increase in the international trade of the wood products (*e.g.*, wood packaging, tree logs, and wood chips), contribute to the introduction and spread of insect pests to uninfested geographic regions with *Eucalyptus* plantations (Andrade et al., 2016; Almeida et al., 2018; Meurisse et al., 2019; Tomé et al., 2021).

The Eucalyptus snout beetle, *Gonipterus platensis* Marelli (Coleoptera: Curculionidae), is native to Australia and is the most widely distributed defoliating beetle of *Eucalyptus* in the world (Hurley et al., 2016). This insect was reported in the Brazilian states of Espírito Santo, Rio Grande do Sul, Santa Catarina, Paraná, and São Paulo with higher damage in plantations of the latter two states (Wilcken et al., 2008; Souza et al., 2016), especially in *Eucalyptus grandis* W. Hill. × *Eucalyptus urophylla* S.T. Blake hybrids (Souza et al., 2016).

*Gonipterus platensis* adults feed on the young and middle-aged leaves and on soft bark of twigs, while its larvae feed exclusively on shoot tips and young leaves (Souza et al., 2016). High infestations of *G. platensis* cause dieback of shoot tips, which may induce the development of epicormic shoots and severe defoliation of the upper third of tree canopy. Sequential defoliations may result in growth of multiple leader shoots and mortality of branches or even trees (Tooke, 1955). Damage by *G. platensis* is low in Australia because of the natural resistance of *Eucalyptus* species and the suppression of this pest by a diversity of natural enemies (Valente et al., 2017; Valente et al., 2019; Afonso et al., 2019). However, this insect causes severe damage on exotic *Eucalyptus* species in some African, American, and European countries (Reis et al., 2012; Souza et al., 2016; Valente et al., 2018; Schröder et al., 2020), resulting in a constant search for alternatives to control this pest (Nascimento et al., 2017; Damascena et al., 2020; Schröder et al., 2021).

The susceptibility of *Eucalyptus* genotypes to *Gonipterus* spp. Schoenherr varies according to the *Eucalyptus* sections (taxonomic division of subgenus), species, and hybrids. The *Symphormitus* subgenus includes about 470 species divided into 11 sections according to their taxonomic and molecular characteristics, of which the *Exsertaria*, *Latoangulatae*, and *Maidenaria* sections represent about 90% of the area planted with *Eucalyptus* in the world (Nicolle & Jones, 2018; Scanavaca Junior & Garcia, 2021). High populations of the *G. platensis* and extensive defoliation are more common on *Eucalyptus* species of the *Maidenaria* section (Garcia et al., 2019; Gonçalves et al., 2019). *Gonipterus platensis* in its region of origin infests species of the *Maidenaria* section including *Eucalyptus dalrympleana* Maiden, *Eucalyptus globulus* Labill., *Eucalyptus nitens* (H. Deane & Maiden) Maiden, *Eucalyptus ovata* Labill., *Eucalyptus rubida* H. Deane & Maiden, and *Eucalyptus viminalis* Labill (Mapondera et al., 2012; Garcia et al., 2019). This pest also infests *Eucalyptus* species of different sections in countries where it has been introduced. In the Iberian Peninsula, species of the *Maidenaria* section are defoliated into variable levels, but those of the *Latoangulatae* section, like *Eucalyptus saligna* Sm., are only lightly defoliated (Reis et al., 2012; Valente et al., 2018; Gonçalves et al., 2019). In Chile, severe defoliation on *E. globulus* (Maidenaria section) and on species of the *Exsertaria* section such as *Eucalyptus camaldulensis* Dehnh. has been reported (Lanfranco & Dungey, 2001). In Brazil, species of the sections *Exsertaria*, *Latoangulatae* and *Maidenaria* are largely planted but *Eucalyptus dunnii* Maiden, *E. globulus* and *E. viminalis* (*Maidenaria* section) are the most damaged by *G. platensis,* which also damages other species such as *Eucalyptus saligna* (var. *protusa*) (*Latoangulatae* section) and the hybrids *E. grandis* × *E. urophylla* (=HGU) and *E. grandis* × *E. dunnii* (Wilcken et al., 2008; Souza et al., 2016).

The losses in wood yield by *G. platensis* in Brazil varies within plant genetic materials from a mean annual increment (MAI) reduction of 10.0% on *E. grandis* to 42.8% on *E. grandis* × *E. dunnii* hybrids (Souza et al., 2016). In 2003, *G. platensis* damaged around 50,000 ha of a HGU clonal plantation in Espírito Santo State with its aggressiveness associated with the high susceptibility of this plant material (Wilcken et al., 2008).

Chemical, nutritional and/or morphological differences in leaves between *Eucalyptus* genotypes, such as secondary compounds, leaf waxes, nitrogen and tannin levels affect the host selection (Gripenberg et al., 2010) and the insect development (Ohmart & Edwards, 1991; Koul , 2008; Behmer, 2009; Gherlenda et al., 2016), which may explain the susceptibility of *Eucalyptus* genotypes to *G. platensis*. The selection of host plants by *G. platensis* is influenced by the emission of volatiles from green leaves and terpenes, such as terpenol 1,8-cineole (Bouwer et al., 2014; Branco et al., 2019), which is more concentrated in *Maidenaria* species susceptible to *G. platensis* than *Latoangulatae* species.

Species of the *Latoangulatae* section are the most planted *Eucalyptus* in Brazil and their susceptibility to *G. platensis* is poorly known, especially in tropical areas, for lack of information on the suitability of these *Eucalyptus* species to this insect. Field observation, host plant response, and insect pest performance are methods used to evaluate host-plant suitability to phytophagous insects (Donatelli et al., 2017). The field observation method integrates the host plant response and insect pest performance with environmental conditions in natural and uniform outbreaks over experimental areas where uncontrolled environments can lead to experimental errors (Sallé et al., 2017). The host plant response in the field is difficult to evaluate for *Gonipterus - Eucalyptus* interactions because this insect feeds on trees from one to six years old, with reduced possibilities of being monitored in field conditions. Therefore, the insect pest performance in laboratory, under controlled conditions, allows a more adequate assessment of biological parameters at all stages of the insect than that on field studies.

The objective of this work was to evaluate the development, reproduction and survival of *G. platensis* fed with leaves of *E. camaldulensis*, *E. grandis*, *E. urophylla*, and of the hybrids HGU ‘H13’ and ‘VR3748’ of *Eucalyptus grandis* × *E. urophylla* under controlled conditions, and to determine the susceptibility of these plants to this insect pest. Our hypothesis was that commercial *Eucalyptus* species/clones cultivated in Brazil affect differently the development, reproduction, and survival of *G. platensis*. The information can be used to manage this pest, avoiding extensive plantations with susceptible *Eucalyptus* species, and reducing the risks of population outbreaks in commercial plantations.

## Material & Methods

### Insect

The rostrum of *G. platensis* adults is short with an ochraceous brown and often reddish colour with 5.7 to 8.9 mm long for males and 7.5 to 8.9 mm for females (Rosado-Neto & Marques, 1996). Females lay eggs forming capsules covered by a dark secretion mainly composed of excrements. *Gonipterus platensis* has four larval instars. The colour of the body of the first and second instar larvae is yellow and the others with three dark-green lateral stripes on the dorsum, which distinguish this species from *G. pulverulentus*, without stripes on the body (Rosado-Neto & Marques, 1996). *Gonipterus platensis* larvae bury themselves in the soil to pupate in a pupal chamber made of sand and fluids secreted by the larvae.

### Insect collection

The experiment was carried out at the Biological Control of Forest Pests Laboratory (LCBPF) at the Department of Plant Protection of the School of Agricultural Sciences (FCA) at the São Paulo State University (UNESP) in Botucatu, São Paulo State, Brazil. *Gonipterus platensis* adult females and males were manually collected in a field stand of *E. grandis* × *E. urophylla* clonal plants in the Espírito Santo State, Brazil, placed in 1 L plastic containers and taken to the LCBPF. At arrival in the laboratory, the insects were kept in rearing cages (40 cm long ×80 cm high ×45 cm wide) at 26 ± 1 °C, 70 ± 10% RH and 14:10 h (L:D) photoperiod receiving fresh HGU shoots as a food source (Wilcken et al., 2008).

### Insect rearing

*Gonipterus platensis* egg capsules were manually collected daily from the rearing cages and individually transferred to glass Petri dishes (9.0 cm diameter) (one capsule/dish), where they were kept in a biochemical oxygen demand (BOD) incubator chamber (model EL202; EletroLab, São Paulo, Brazil) at 26 °C and 14:10 h (L:D) photoperiod to obtain larvae of this insect. One hundred newly-hatched *G. platensis* larvae were individually placed per transparent, cylindrical plastic container (7 cm high ×4 cm diameter) with a fresh, young leaf of either *E. camaldulensis*, *E. grandis*, *E. urophylla*, or the *E. urophylla* × *E. grandis* hybrids ‘VR3748’ and ‘H13’ with each plant genotype representing a treatment. *Gonipterus platensis* larvae were assessed daily until the pre-pupa stage. The pre-pupae were individualized in plastic containers (7 cm high ×4 cm diameter) on surface of a fine autoclaved sand layer (40 ml of sand and 2.5 cm deep) as a pupation substrate.

The sex of the newly-emerged *G. platensis* adults was identified according to the external morphology of their fifth abdominal sternite (Rosado-Neto & Marques, 1996). Pairs were formed with healthy and vigorous newly-emerged adults and each pair placed per transparent, conical-shaped plastic container (6 cm high ×10 cm upper opening diameter ×8 cm lower opening diameter) covered with a fine-mesh nylon fabric piece for aeration. The *G. platensis* couples received shoots with tender leaves of *Eucalyptus* species or clones as a food source (according to the treatment) and substrate for oviposition. The petiole of these shoots was placed in 2-ml plastic Eppendorf^®^ tubes (Hamburg, Germany) filled with water + gel (Hydroplan-EB–0.25%) to keep them fresh and suitable for insect feeding and oviposition. The shoots consumed were replaced daily by fresh ones. Non-mated *G. platensis* adults were individualized in 500 mL plastic containers, receiving a *Eucalyptus* leaf daily according to the treatment, and used to estimate the adult longevity.

### Assessed parameters

The periods of larva to adult; complete lifespan (egg + larva + pupa + adult); pre- and oviposition (days) and egg incubation (days) were evaluated; besides viability of larva to adult and pupal stage (%); number, duration (days) and viability of each instar; adult (males and females separately and combined) longevity (days); numbers of egg capsules/female and of larvae hatched/egg capsule; and viability of eggs/egg capsule (%). The number and duration of each instar was daily assessed using a stereomicroscope (Nikon SMZ645) when needed to examine the presence of exuviae and/or head capsules released on the *Eucalyptus* leaf or containers’ inner surfaces. The pupal life stage period (pre-pupa + pupa) was determined as the period between larva digging in the sand and adult emergence. The pre-pupal and pupal life stage periods were expressed as pupal life stage period because of the impossibility of identifying these stages separately without destructive sampling of pupal chamber.

The reproductive parameters of *G. platensis* were evaluated with oviposition obtained from the laboratory rearing colony. The leaves with egg capsules were cut from the shoots and each egg capsule placed in a plastic Petri dish (9.0 cm diameter), kept in a BOD incubator chamber (EletroLab model EL202) at 26 °C and 14:10 h (L:D) photoperiod until all viable larvae hatched. The number of *G. platensis* couples varied between treatments because of differences in larval and pupal viability and in the number of adults obtained. The complete lifespan, obtained by calculating the median duration (*i.e.*, period in which 50% of the individuals completed every stage), was determined per stage and after the death of all individuals.

### Experiment design and statistics

The experiment was arranged in a complete randomize design (CRD) with 100 insects (replicates) per *Eucalyptus* species or hybrid. Egg, larval, pre-pupa, pupa and adult data were collected for each insect. All data were submitted to analysis of normality of residuals and homogeneity of variances (Univariate Procedure; Statistical Analysis System SAS^®^, 2001). The data with normal distribution of residuals: period of each instar and larva stage (days), pupal period (days), larva to adult period (days), adult longevity (days), pre- and oviposition periods (days), and number of egg capsules/female were subjected to an Analysis of Variance (one way ANOVA), with the means compared by Tukey’s range test (Tukey, 1949), while those that did not follow a normal distribution: viability of larval stage (%), pupal stage (%), and larva to adult (%), and egg viability/egg capsule (%) subjected to the Kruskal-Wallis test (Kruskal & Wallis, 1952), with the means compared by the Nemenyi test (Nemenyi, 1963). The data of viability was previously transformed into arcsine $\sqrt{x}\div 100$ to homogenize the data variance (Haddad & Vendramim, 2000). The survival data were analysed for the larval stage and larva to adult periods (Lifest Procedure; Statistical Analysis System SAS , 2001). The differences per biological parameter of *G. platensis* among *Eucalyptus* host genotypes were obtained comparing the means using the Savage test (Savage, 1972). Data of percentage of *G. platensis* in the larval stage during the time (days) until the transformation into pupa and adult with different *Eucalyptus* species and hybrids were calculated to verify the uniformity and time to transformation in these stages. The significance level was 0.05.

## Results

### Instar periods and larval stage viability

**Figure 1:**
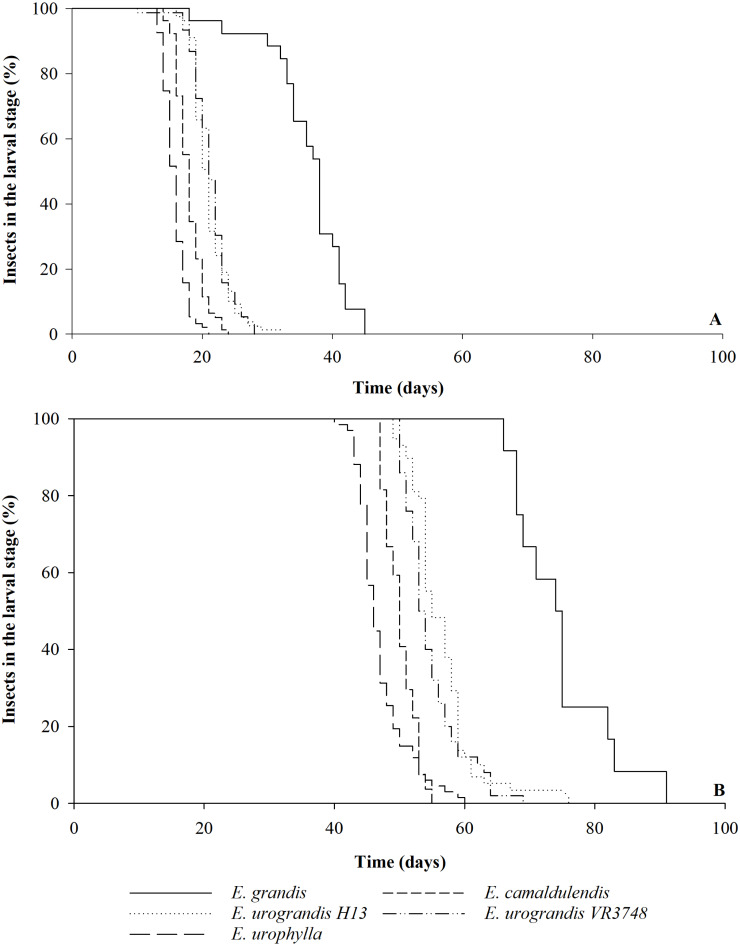
Larval stage and larva to adult period of *Gonipterus platensis* on *Eucalyptus* hosts. Percentage of *Gonipterus platensis* in the larval stage during the time (days) until the transformation into pupa (A) and adult (B) on different *Eucalyptus* (*E.*) species and hybrids at 26 °C and 14:10 h (L:D) photoperiod.

The duration of each instar of *G. platensis* varied according to the *Eucalyptus* species or hybrids (Savage test *χ*^2^ = 472.96; Pr >*χ*^2^ 0.0001, Table 1). The durations of first and second instars and of larval stage were shorter on *E. camaldulensis* and *E. urophylla*, the third was shorter on *E. urophylla* and the fourth instar was longer with *E. grandis*. The duration of the larval stage was shorter on *E. urophylla* (15.7 ± 0.2 days) than on *E. camaldulensis* (17.9 ± 0.2 days), HGU ‘H13’ and ‘VR3748’ (20.8 ± 0.4 and 21.3 ± 0.3 days, respectively), and *E. grandis* (37.6 ± 0.8 days) (Table 1). The last one also showed smaller uniformity and longer time for complete transformation from the larvae population to pupae (Fig. 1A). The larval stage viability was higher on *E. urophylla* (93.0 ± 2.6%), *E. camaldulensis* (78.0 ± 4.9%), HGU ‘H13’ (79.0  ± 5.6%), and HGU ‘VR3748’ (76.0 ± 4.9%) than on *E. grandis* (24.0 ± 4.0%) (Table 1; Fig. 1A).

### Pupal period and viability

The pupal stage period of *G. platensis* was shorter in *E. urophylla* (31.4 ± 0.4 days) and *E. camaldulensis* (32.6 ± 0.4 days) than on HGU ‘VR3748’ (33.5 ± 0.5), ‘H13’ (35.3 ± 0.5 days) and *E. grandis* (37.5 ± 1.7 days). The viability of the pupal stage was higher in the hybrid ‘H13’ (74.7 ± 3.1%), *E. urophylla* (74.2 ± 6.0%) and the hybrid ‘VR3748’ (65.8 ± 3.3%) than in *E. grandis* (50.0 ± 3.4%) and *E. camaldulensis* (33.3 ± 6.5%) (Table 1).

### Larva to adult period and viability

The larva to adult period varied between the *Eucalyptus* genotypes (Savage test *χ*^2^ = 189.59; Pr >*χ*^2^ 0.0001) was shorter in *E. urophylla* (47.2 ± 0.9 days) and *E. camaldulensis* (50.5 ± 0.5 days) than in the hybrids ‘VR3748’ (54.8 ± 0.6 days) and ‘H13’ (56.1 ± 0.7 days) and *E. grandis* (75.1 ± 2.1 days) (Table 1). The last one also showed smaller uniformity and longer time for complete transformation from the larvae population to adult (Fig. 1B). The larva to adult viability was higher in *E. urophylla* (69.0 ± 3.1%) and the HGU ‘H13’ (59.0 ± 8.1%) and ‘VR3748’ (50.0 ± 4.5%) than in *E. camaldulensis* (27.0 ± 4.2%) and *E. grandis* (12.0 ± 3.1%) (Table 1). A total of 50% of the adults emerged within a 46-day period with *E. urophylla*, compared to a 50, 53, 55, and 74-day periods for *E. camaldulensis*, the hybrid ‘VR3748’, the hybrid ‘H13’, and *E. grandis*, respectively (Fig. 1B).

**Table 1:** Duration, viability of larval and pupal stages of *Gonipterus platensis* on *Eucalyptus hosts*. Duration (days) of the first (I1), second (I2), third (I3) and fourth (I4) instars and of the larval (LS) and pupal stage (PS), and larva to adult (LA) (mean  ± SE), amplitudes (days) (A), and viabilities (V, %) of *Gonipterus platensis* (Coleoptera: Curculionidae) on *Eucalyptus camaldulensis*, *Eucalyptus grandis*, *Eucalyptus urophylla*, and on the *E. grandis* × *E. urophylla* hybrids ‘VR3748’ and ‘H13’ at 26 °C and 14:10 h (L:D) photoperiod.

Parameters	*E. camaldulensis*	*E. grandis*	‘VR3748’	‘H13’	*E. urophylla*
I1^1^	4.7 ± 0.2c	9.9 ± 0.5a	5.8 ± 0.1b	5.2 ± 0.1bc	4.5 ± 0.1c
I2^1^	3.6 ± 0.1d	9.1 ± 0.3a	4.9 ± 0.2bc	5.7 ± 0.2ab	3.9 ± 0.2cd
I3^1^	5.0 ± 0.3b	10.0 ± 0.2a	5.2 ± 0.2b	5.0 ± 0.2b	3.6 ± 0.1c
I4^1^	4.4 ± 0.3b	11.5 ± 0.5a	5.0 ± 0.2b	5.0 ± 0.2b	4.3 ± 0.1b
LS^1^	17.9 ± 0.2c	37.6 ± 0.8a	21.3 ± 0.3b	20.8 ± 0.4b	15.7 ± 0.2d
V(%)^2^	78.0 ± 4.9a	24.0 ± 4.0b	76.0 ± 4.9a	79.0 ± 5.6a	93.0 ± 2.6a
PS^1^	32.6 ± 0.4bc	37.5 ± 1.7a	33.5 ± 0.5ab	35.3 ± 0.5a	31.4 ± 0.4c
V(%)^2^	33.3 ± 6.5b	50.0 ± 3.4b	65.8 ± 3.3a	74.7 ± 3.1a	74.2 ± 6.0a
LA^1^	50.5 ± 0.5c	75.1 ± 2.1a	54.8 ± 0.6b	56.1 ± 0.7b	47.2 ± 0.9c
A	47–54	66–91	50–69	49–76	43–60
V(%)^2^	27.0 ± 4.2b	12.0 ± 3.1b	50.0 ± 4.5a	59.0 ± 8.1a	69.0 ± 3.1a

**Notes.**

Means followed by the same letter, per row, do not differ by the ^1^Nemenyi and ^2^Tukey’s range tests, both at *p* < 0.05.

### Adult complete lifespan and longevity

The adult (females + males) complete lifespan was longer in *E. urophylla* (223.9 ± 11.2 days) than in the hybrids ‘VR3748’ and ‘H13’ (171.3 ± 14.01 and 146.1 ± 10.6 days, respectively), *E. grandis* (61.9 ± 16.9 days), and *E. camaldulensis* (75.2 ± 8.4 days). The longevity of adult females was longer in *E. urophylla* (283.3 ± 18.3 days) than in *E. camaldulensis* (103.8 ± 12.6 days) and *E. grandis* (65.3 ± 27.5 days) and that of males longer in *E. urophylla* (175.8 ± 10.5 days), ‘VR3748’ (149.8 ± 11.6 days) than in ‘H13’ (127.4 ± 11.0 days), with the second lowest duration on *E. camaldulensis* (68.1 ± 9.4 days) and the lowest longevity in *E. grandis* (60.8 ± 21.4 days) (Table 2).

### Reproduction

The reproductive parameters of adult females of *G. platensis* varied with *Eucalyptus* host. The pre-oviposition was longer (55 days) in *E. camaldulensis* than in *E. urophylla* (28.7 ± 0.9 days) and the hybrid ‘H13’ (33.2 ± 1.3 days) with intermediate period with the ‘VR3748’ (30.1 ± 1.2 days). The insect laid no eggs on *E. grandis* and only a single egg capsule on *E. camaldulensis* (Table 3).

**Table 2:** Lifespan and longevity of *Gonipterus platensis* on *Eucalyptus hosts*. Complete lifespan (CL, days), longevity of adults (males + females), and females and males separately (mean  ± SE) of *Gonipterus platensis* (Coleoptera: Curculionidae) on the hosts *Eucalyptus camaldulensis*, *Eucalyptus grandis*, *Eucalyptus urophylla*, and on the *E. grandis* × *E. urophylla* hybrids ‘VR3748’ and ‘H13’ at 26 °C and 14:10 h (L:D) photoperiod.

*Eucalyptus* hosts	CL^1^	Longevity (days)		
		Males + Females	Females	Males
*E. camaldulensis*	125.7 ± 8.6d	75.2 ± 8.4c	103.8 ± 12.6b	68.1 ± 9.4cd
*E. grandis*	137.0 ± 16.9cd	61.9 ± 16.9c	65.3 ± 27.5b	60.8 ± 21.4d
‘VR3748’	226.1 ± 14.1b	171.3 ± 14.01b	248.0 ± 43.2ab	149.8 ± 11.6ab
‘H13’	202.2 ± 10.7bc	146.1 ± 10.6b	195.3 ± 21.2ab	127.4 ± 11.0bc
*E. urophylla*	271.1 ± 11.2a	223.9 ± 11.2a	283.3 ± 18.3a	175.8 ± 10.5a

**Notes.**

1 Means followed by the same letter, per column, do not differ by the Nemenyi test at *p* < 0.05.

**Table 3:** Pre-oviposition and oviposition periods of *Gonipterus platensis* on *Eucalyptus hosts*. Pre- and oviposition periods (days, mean  ± SE), and number of replications (n, females) of *Gonipterus platensis* (Coleoptera: Curculionidae) on the hosts *Eucalyptus camaldulensis*, *Eucalyptus grandis*, *Eucalyptus urophylla*, and on the *E. grandis* × *E. urophylla* hybrids ‘VR3748’ and ‘H13’ at 26 °C and 14:10 h (L:D) photoperiod.

*Eucalyptus* hosts	Pre-oviposition^1^	Oviposition^2^	*n*
*E. camaldulensis*	55.0	4.0	1
*E. grandis*	^*^	^*^	^*^
‘VR3748’	30.1 ± 1.2ab	114.6 ± 23.3a	10
‘H13’	33.2 ± 1.3a	98.6 ± 31.2a	12
*E. urophylla*	28.7 ± 0.9b	166.0 ± 19.8a	18

**Notes.**

Means followed by the same letter, per column, do not differ by the ^1^Nemenyi and ^2^Tukey’s range tests, both at *p* < 0.05.

* Insufficient data for analysis.

The *G. platensis* adult females began oviposition on host shoot tips and leaves up to the following day after copulation. The oviposition period was similar among *E. urophylla* (166.0 ± 19.8 days) and the hybrids HGU ‘VR3748’ (114.6 ± 23.3 days) and ‘H13’ (98.6 ± 31.2 days) (Table 3).

The number of egg capsules/female was higher in the hybrid ‘VR3748’ (152.3 ± 29.7 egg capsules) and *E. urophylla* (98.0 ± 14.19 egg capsules) than in ‘H13’ (36.2  ± 6.02 egg capsules). The egg incubation period was similar among *Eucalyptus* hosts, between 7.1 to 7.3 days. The egg viability/egg capsule was higher in *E. urophylla* (80.3  ± 3.99%), than on the hybrid ‘H13’ (56.4 ± 6.92%) and intermediate values in ‘VR3748’ (73.2 ± 7.02%). The total number of larvae/egg capsules was higher in ‘VR3748’ (228.30 ± 39.5 larvae) and in *E. urophylla* (177.7  ± 31.1 larvae) than in ‘H13’ (40.8 ± 9.8 larvae) (Table 4).

**Table 4:** Reproductive parameters of *Gonipterus platensis* on *Eucalyptus hosts*. Number of egg capsules/female (ECF), egg incubation period (EI in days), number of larvae hatched (LH), and viability of eggs/egg capsule (VE/EC in, %) (mean  ± SE) of *Gonipterus platensis* (Coleoptera: Curculionidae) on the hosts *Eucalyptus camaldulensis*, *Eucalyptus grandis*, *Eucalyptus urophylla*, and on the *E. grandis* × *E. urophylla* hybrids ‘VR3748’ and ‘H13’ at 26 °C and 14:10 h (L:D) photoperiod.

*Eucalyptus* hosts	ECF^1^	EI^1^	LH^2^	VE/EC (%)^2^
*E. camaldulensis*	1.0	7.3	4.0	^*^
*E grandis*	^*^	^*^	^*^	^*^
‘VR3748’	152.3 ± 29.7a	7.2 ± 0.08a	228.3 ± 39.5a	73.2 ± 7.02ab
‘H13’	36.2 ± 6.02b	7.1 ± 0.10a	40.8+9.8b	56.4 ± 6.92b
*E. urophylla*	98.0 ± 14.19a	7.2 ± 0.03a	177.7 ± 31.1a	80.3 ± 3.99a

**Notes.**

Means followed by the same letter, per column, do not differ by the ^1^Nemenyi and ^2^Tukey’s range tests, both at *p* < 0.05.

* Insufficient data for analysis.

## Discussion

The environmental conditions in the *Eucalyptus* plantations in South and Southeast Brazil are similar to those of the *Gonipterus* distribution in Australia, which are a humid subtropical zone with a temperate climate and hot or temperate summers (Crosbie et al., 2012; Mapondera et al., 2012; Alvares et al., 2013). The damage by *G. platensis* was low in these areas up to 2012 and increased from the end of 2012 (Souza et al., 2016), probably because of the replacement of *E. grandis* by more productive HGU clones (C.F.W. personal information, 2022). The *G. platensis* survival, development and reproduction differ among the *Eucalyptus* species and hybrids with *E. urophylla* being the most suitable host for this insect, the HGU ‘H13’ and ‘VR3748’ intermediate, and *E. grandis* and *E. camaldulensis* the least suitable ones. Higher viability, adult longevity, and fecundity of *G. platensis* on *E. urophylla* indicate that species of *Latoangulatae* section can be suitable hosts for this insect. Nevertheless, the susceptibility of *E. saligna*, another species of the *Latoangulatae*, to *G. platensis* is low (Gonçalves et al., 2019). This suggests different susceptibilities among species within this section. However, *E. grandis* and *E. camaldulensis* were poor suitable hosts, and feeding on leaves of these species reduced survival, adult longevity and fecundity of this beetle. *Eucalyptus urophylla* is native to islands of the Indonesian archipelago and Timor (Hodge & Dvorak, 2015) and is not one of the native hosts of *G. platensis* in its native region, Tasmania (Australia) (Mapondera et al., 2012). This fact indicates possibilities of *G. platensis* to adapt to novel hosts as reported for *Gonipterus* sp. n.2 (Newete, Oberprieler & Byrne, 2011) and other insects. *Paropsisterna bimaculata* Olivier (Coleoptera: Chrysomelidae) become a pest of *E. nitens* (*Maidenaria* section) after this host was introduced in Tasmania. In the past, the insect was thought to be host-specific for species of the subgenus *Eucalyptus* (de Little & Madden, 1976; Paine, Steinbauer & Lawson, 2011). The oviposition site selection by *P. bimaculata* females depends on host morphological characteristics because the insect holds the leaf edge while ovipositing and plant kairomones seem to be of low importance (Howlett & Clarke, 2003). On the other hand, *Anoplognathus montanus* Macleay and *A. pallidicollis* Blanchard (Coleoptera: Scarabaeidae) that are considered host specific to eucalyptus, can also feed on *Schinus molle* L. (Anacardiaceae), an exotic plant from South America. This unusual feeding behavior was explained by the presence of similar monoterpenes in both host plants (Steinbauer & Wanjura, 2002).

The shorter larval stage duration on *E. urophylla* than on other species, possibly, because it has better nutritional value for the larval development of *G. platensis*. Similarly, *E. globulus* foliage (*Maidenaria* section) is considered one the most preferred host to *G. platensis* in Spain, with a shorter duration of the larval stage (22.1 days) than other species (Cordero-Rivera & Santolamazza-Carbone, 2000; Santolamazza-Carbone, Rodríguez-Illamamola & Cordero Rivera, 2006; Gonçalves et al., 2019). The intermediate larval stage duration of *G. platensis* on the hybrids HGU ‘H13’ (20.8 ± 0.4 days) and ‘VR3748’ (21.3  ± 0.3 days) is similar to that of this insect on *E. globulus* (Santolamazza-Carbone, Rodríguez-Illamamola & Cordero Rivera, 2006). The low larval stage viability on *E. grandis* indicates that this species is inadequate for *G. platensis* larval development.

The shorter pupal period on *E. urophylla* indicates the quality of this *Eucalyptus* species for *G. platensis*. Higher survival and shorter development period indicate better host quality, as the development duration is extended in inadequate hosts to increase the food intake, especially when the nutrient balance becomes sub-optimal (Chapman, 2013; Bawin et al., 2016). In that case, *E. urophylla* has both parameters, but a short development period and lower survival of the pupal stage of this insect were found on *E. camaldulensis*. The higher pupal viability with *E. urophylla*, HGU ‘H13’ and ‘VR3748’ indicates again the quality of *E. urophylla* (also presented in the HGUs) for *G. platensis* development and its mortality in the pupal stage reflecting the conditions to which its larva was exposed (Nestel et al., 2016), with poorer diets increasing pupa mortality (Mohammadzadeh & Izadi, 2018). The lower pupa viability of *G. platensis* on *E. camaldulensis* may be due to chemical and/or morphological differences of leaves between *Eucalyptus* genotypes such as secondary compounds, leaf waxes, nitrogen levels, and tannins (Ohmart & Edwards, 1991; Gherlenda et al., 2016) affecting insect development. *Eucalyptus camaldulensis* was also inadequate to *Gonipterus pulverulentus* Lea with lower food conversion efficiency and larval weight among the evaluated hosts, indicating worse larval fitness (Riquelme Virgala et al., 2018).

The shorter larva to adult period and higher survival rates on *E. urophylla* indicate that this species is a suitable food source to *G. platensis* (Bawin et al., 2016). Chemical analysis to identify which compounds are responsible for suitable food sources need to be further investigated. In contrast, longer development period in inadequate host plants, as on *E. grandis,* can increase larval exposure to natural enemies and mortality of *G. platensis* in the field, according to the slow-growth high-mortality hypothesis (Clancy & Price, 1987; Uesugi, 2015). Variations in the development period of *G. platensis* among *Eucalyptus* host species and hybrids suggest an effect of the food quality, as generally reported for insect herbivores (Behmer, 2009). This development period reflects nutritional, morphological, chemical composition, and plant-defence differences between host plants (Paine, Steinbauer & Lawson, 2011; Malishev & Sanson, 2015; Oates et al., 2015; Santadino et al., 2017).

The oviposition period was similar between *E. urophylla* and HGUs hybrids, the only hosts with oviposition by *G. platensis*. The higher numbers of egg capsules/female on the ‘VR3748’ and *E. urophylla* are related to host choice with herbivorous insects choosing those with better conditions for survival and development of their progeny (Gripenberg et al., 2010). The number of egg capsules of *Gonipterus* sp. n.2 was also high on *E. urophylla* in South Africa (Newete, Oberprieler & Byrne, 2011). This species laid eggs on *E. grandis* and *E. camaldulensis* (Newete, Oberprieler & Byrne, 2011) whereas *G. platensis* did not, showing different oviposition preferences between these two *Gonipterus* species. The emission of volatile organic compounds, such as green leaf volatiles and terpenes may influence host plant selection by *G. platensis* (Bouwer et al., 2014; Branco et al., 2019). The terpenoid 1,8-cineole is probably responsible for the attractiveness and its metabolization by *G. platensis* results in the production of hydroxylated derivatives that are likely to act as sex pheromones for this beetle (Branco et al., 2020). This terpenoid is highly abundant in the leaf oil composition of *Maidenaria* species susceptible to *G. platensis,* like *E. dunnii* (43.67%), *E. globulus* (69.10%), *E. nitens* (47.9%), and *E. viminalis* (63.73%) and with a low percentage in *Latoangulatae* species with low damage by *G. platensis* like *E. grandis* (0.45%), and *E. saligna* (0.11%) (Boland, Brophy & House, 1991; Batista-Pereira et al., 2006). The high percentage of 1,8-cineole (53.11%) in the *E. urophylla* (*Latoangulatae* section) (Batista-Pereira et al., 2006), makes this species attractive to *G. platensis*, similar to *Maidenaria* species.

The unsuitability of *E. camaldulensis* and *E. grandis* to *G. platensis*, compared to the longer pre-oviposition period, lower number of egg capsules/female, and egg viability with the hybrid ‘H13’ indicate the potential of those species for planting as a management strategy for this pest. These plant materials can be used in separate stands on most of the available area or in mixed ones in mosaic or using *E. grandis* as a barrier in a mosaic landscape (Forrester, Bauhus & Khanna, 2004; Martins et al., 2014).

## Conclusions

The shortest egg-to-adult development period, greatest longevity, reproduction, and viability of *E. urophylla* indicate the suitability of this plant for *G. platensis*. The intermediate values of the evaluated parameters for *G. platensis* that fed on the HGU ‘VR3748’ and ‘H13’ indicate that these plants are also appropriate to this pest. The egg-to-adult development period was shorter and the larval stage viability high for *G. platensis* on *E. camaldulensis*, but the low larva to adult viability and reproduction impaired the establishment of *G. platensis* on *E. camaldulensis*. The longest period and the lowest viability of the larval stage and reproduction of *G. platensis* on *E. grandis* indicated this is the least suitable host tested for this insect.

The insect performance method utilized to assess the suitability of *Eucalyptus* genotypes to *G. platensis*, allowed to evaluate how host quality affects the survival, development, and reproduction of this insect, and can be replicated for other pest species of economic importance.

The information can be used to manage *G. platensis*, by avoiding extensive plantations with susceptible species, and reducing the risks of population outbreaks in commercial plantations.

##  Supplemental Information

10.7717/peerj.13698/supp-1 Supplemental Information 1 Raw Data Parameters analyzed and described in the article.

## Additional Information and Declarations

### Data Availability

The following information was supplied regarding data availability: The raw data are available in the Supplemental File and at Zenodo: DOI Oliveira, Nádia Cristina, Ribeiro, Murilo Fonseca, Ottati, Ângelo Luiz Tadeu, Tavares, Wagner de Souza, Serrão, José Eduardo, Zanuncio, José Cola, Zanetti, Ronald, & Wilcken, Carlos Frederico. (2022). Raw Data Survival, Development and Reproduction of Gonipterus platensis on Eucalyptus genotypes [Data set]. Zenodo. 10.5281/zenodo.6672350.

## References

[ref-1] Afonso C, Valente C, Gonçalves CI, Reis A, Cuenca B, Branco M (2019). *Anagonia* sp. (Diptera: Tachinidae), potential biocontrol agent of *Gonipterus platensis* (Coleoptera: Curculionidae) in the Iberian Peninsula. IOBC-WPRS Bulletin.

[ref-2] Almeida KEG, Silva JGS, Silva IMA, Costa AL, Laia ML (2018). Ecophysiological analysis of *Eucalyptus camaldulensis* (Dehnh) submitted to attack from *Thaumastocoris peregrinus* (Carpintero & Dellape). Revista Árvore.

[ref-3] Alvares CA, Stape JL, Sentelhas PC, de Moraes Gonçalves JL, Sparovek G (2013). Köppen’s climate classification map for Brazil. Meteorologische Zeitschrift.

[ref-4] Andrade JGF, Sá VGM, Lodi S, Godoy BS (2016). Age of *Eucalyptus urograndis* plantations and occurrence of pest insects. Revista Árvore.

[ref-5] Batista-Pereira LG, Fernandes JB, Corrêa AG, Silva MFGF, Vieira PC (2006). Electrophysiological responses of eucalyptus brown looper *Thyrinteina arnobia* to essential oils of seven *Eucalyptus* species. Journal of the Brazilian Chemical Society.

[ref-6] Bawin T, Dujeu D, de Backer L, Francis F, Verheggen FJ (2016). Ability of *Tuta absoluta* (Lepidoptera: Gelechiidae) to develop on alternative host plant species. Canadian Entomologist.

[ref-7] Behmer ST (2009). Insect herbivore nutrient regulation. Annual Review of Entomology.

[ref-8] Boland DJ, Brophy JJ, House APN (1991). Eucalyptus leaf oils: use, chemistry, distillation and marketing.

[ref-9] Bouwer MC, Slippers B, Wingfield MJ, Rohwer ER (2014). Chemical signatures affecting host choice in the *Eucalyptus* herbivore, *Gonipterus* sp. (Curculionidae: Coleoptera). Arthropod-Plant Interactions.

[ref-10] Branco S, Mateus EP, da Silva MDRG, Mendes D, Pereira MMA, Schütz S, Paiva MR (2020). Identification of pheromone candidates for the eucalyptus weevil, *Gonipterus platensis* (Coleoptera, Curculionidae). Journal of Applied Entomology.

[ref-11] Branco S, Mateus EP, da Silva MDRG, Mendes D, Rocha S, Mendel Z, Schütz S, Paiva MR (2019). Electrophysiological and behavioural responses of the Eucalyptus weevil, *Gonipterus platensis*, to host plant volatiles. Journal of Pest Science.

[ref-12] Chapman RF (2013). The insects structure and function.

[ref-13] Clancy KM, Price PW (1987). Rapid herbivore growth enhances enemy attack: sublethal plant defenses remain a paradox. Ecology.

[ref-14] Cordero-Rivera A, Santolamazza-Carbone S (2000). The effect of three species of *Eucalyptus* on growth and fecundity of the Eucalyptus snout beetle (*Gonipterus scutellatus*). Forestry.

[ref-15] Crosbie RS, Pollock DW, Mpelasoka FS, Barron OV, Charles SP, Donn MJ (2012). Changes in Köppen-Geiger climate types under a future climate for Australia: hydrological implications. Hydrology and Earth System Sciences.

[ref-16] Damascena AP, Carvalho VR, Ribeiro MF, Horta AB, Monteiro de Castroe Castro B, Zanuncio AJV, Wilcken CF, Zanuncio JC, Wilcken SRS (2020). *Steinernema diaprepesi* (Rhabditida: Steinernematidae) parasitizing *Gonipterus platensis* (Coleoptera: Curculionidae). Royal Society Open Science.

[ref-17] de Little DW, Madden JL (1976). Host preference in the Tasmanian eucalypt defoliating Paropsini (Coleoptera: Chrysomelidae) with particular reference to *Chrysophtharta bimaculata* (Olivier) and *C*. agricola (Chapuis). Australian Journal of Entomology.

[ref-18] Donatelli M, Magarey RD, Bregaglio S, Willocquet L, Whish JPM, Savary S (2017). Modelling the impacts of pests and diseases on agricultural systems. Agricultural Systems.

[ref-19] FAO (2020). Global Forest Resources Assessment 2020: Main report. Rome.

[ref-20] Forrester DI, Bauhus J, Khanna PK (2004). Growth dynamics in a mixed-species plantation of *Eucalyptus globulus* and *Acacia mearnsii*. Forest Ecology and Management.

[ref-21] Garcia A, Allen GR, Oberprieler RG, Ramos AP, Valente C, Reis A, Franco JC, Branco M (2019). Biological control of *Gonipterus*: Uncovering the associations between eucalypts, weevils and parasitoids in their native range. Forest Ecology and Management.

[ref-22] Gherlenda AN, Moore BD, Haigh AM, Johnson SN, Riegler M (2016). Insect herbivory in a mature *Eucalyptus* woodland canopy depends on leaf phenology but not CO_2_enrichment. BMC Ecology.

[ref-23] Gonçalves CI, Vilas-Boas L, Branco M, Rezende GD, Valente C (2019). Host susceptibility to *Gonipterus platensis* (Coleoptera: Curculionidae) of *Eucalyptus* species. Annals of Forest Science.

[ref-24] Gripenberg S, Mayhew PJ, Parnell M, Roslin T (2010). A meta-analysis of preference-performance relationships in phytophagous insects. Ecology Letters.

[ref-25] Haddad M, Vendramim JD (2000). Comparação de porcentagens observadas com casos extremos de 0 e 100%. Anais da Sociedade Entomológica do Brasil.

[ref-26] Hodge GR, Dvorak WS (2015). Provenance variation and within-provenance genetic parameters in *Eucalyptus urophylla* across 125 test sites in Brazil, Colombia, Mexico, South Africa and Venezuela. Tree Genetics & Genomes.

[ref-27] Howlett BG, Clarke AR (2003). Role of foliar chemistry versus leaf-tip morphology in egg-batch placement by *Chrysophtharta bimaculata* (Olivier) (Coleoptera: Chrysomelidae). Australian Journal of Entomology.

[ref-28] Hurley BP, Garnas J, Wingfield MJ, Branco M, Richardson DM, Slippers B (2016). Increasing numbers and intercontinental spread of invasive insects on eucalypts. Biological Invasions.

[ref-29] IBÁ (2021). Annual Report. https://www.iba.org/datafiles/publicacoes/relatorios/relatorioiba2021-compactado.pdf.

[ref-30] Koul O (2008). Phytochemicals and insect control: an antifeedant approach. Critical Reviews in Plant Sciences.

[ref-31] Kruskal WH, Wallis WA (1952). Use of ranks in one-criterion variance analysis. Journal of the American Statistical Association.

[ref-32] Lanfranco D, Dungey HS (2001). Insect damage in *Eucalyptus*: a review of plantations in Chile. Austral Ecology.

[ref-33] Malishev M, Sanson GD (2015). Leaf mechanics and herbivory defence: how tough tissue along the leaf body deters growing insect herbivores. Austral Ecology.

[ref-34] Mapondera TS, Burgess T, Matsuki M, Oberprieler RG (2012). Identification and molecular phylogenetics of the cryptic species of the *Gonipterus scutellatus* complex (Coleoptera: Curculionidae: Gonipterini). Australian Journal of Entomology.

[ref-35] Martins GS, Moura GPL, Ramalho MAP, Gonçalves FMA (2014). Performance of *Eucalyptus* clones in auto and allocompetition. Silvae Genetica.

[ref-36] Meurisse N, Rassati D, Hurley BP, Brockerhoff EG, Haack RA (2019). Common pathways by which non-native forest insects move internationally and domestically. Journal of Pest Science.

[ref-37] Mohammadzadeh M, Izadi H (2018). Different diets affecting biology, physiology and cold tolerance of *Trogoderma granarium* Everts (Coleoptera: Dermestidae). Journal of Stored Products Research.

[ref-38] Nascimento LI, Soliman EP, Zauza EAV, Stape JL, Wilcken CF (2017). First global record of *Podisus nigrispinus* (Hemiptera: Pentatomidae) as predator of *Gonipterus platensis* (Coleoptera: Curculionidae) larvae and adults. Florida Entomologist.

[ref-39] Nemenyi PB (1963). Distribution-free multiple comparisons. PhD Thesis.

[ref-40] Nestel D, Papadopoulos NT, Pascacio-Villafán C, Righini N, Altuzar-Molina AR, Aluja M (2016). Resource allocation and compensation during development in holometabolous insects. Journal of Insect Physiology.

[ref-41] Newete SW, Oberprieler RG, Byrne MJ (2011). The host range of the Eucalyptus weevil, *Gonipterus scutellatus* Gyllenhal (Coleoptera: Curculionidae), in South Africa. Annals of Forest Science.

[ref-42] Nicolle D, Jones R (2018). A revised classification for the predominantly eastern Australian *Eucalyptus* subgenus Symphyomyrtus sections Maidenaria, Exsertaria, Latoangulatae and related smaller sections (Myrtaceae). Telopea.

[ref-43] Oates CN, Külheim C, Myburg AA, Slippers B, Naidoo S (2015). The transcriptome and terpene profile of *Eucalyptus grandis* reveals mechanisms of defense against the insect pest, *Leptocybe invasa*. Plant Cell Physiology.

[ref-44] Ohmart CP, Edwards PB (1991). Insect herbivory on *Eucalyptus*. Annual Review of Entomology.

[ref-45] Paine TD, Steinbauer MJ, Lawson SA (2011). Native and exotic pests of *Eucalyptus*: a worldwide perspective. Annual Review of Entomology.

[ref-46] Reis AR, Ferreira L, Tomé M, Araujo C, Branco M (2012). Efficiency of biological control of *Gonipterus platensis* (Coleoptera: Curculionidae) by *Anaphes nitens* (Hymenoptera: Mymaridae) in cold areas of the Iberian Peninsula: Implications for defoliation and wood production in *Eucalyptus globulus*. Forest Ecology and Management.

[ref-47] Ribeiro MDdosSB, Rodrigues SA, Ballarin AW (2020). Multivariate association of wood basic density with site and plantation variables in *Eucalyptus* spp. Canadian Journal of Forest Research.

[ref-48] Riquelme Virgala M, Di Silvestro G, Martínez C, Santadino M, Poretti T, Ansa A, Coviella C (2018). Consumo larval y preferencia de oviposición de *Gonipterus pulverulentus* (Coleoptera: Curculionidae) asociados a distintas especies de *Eucalyptus* (Myrtaceae). Bosque.

[ref-49] Rosado-Neto GH, Marques MI (1996). Characteristics of adult, genitalia and immature forms of *Gonipterus gibberus* Boisduval and *G. scutellatus* Gyllenhal (Coleoptera, Curculionidae). Revista Brasileira de Zoologia.

[ref-50] Sallé A, Pointeau S, Bankhead-Dronnet S, Bastien C, Lieutier F (2017). Unraveling the tripartite interactions among the woolly poplar aphid, its host tree, and their environment: a lead to improve the management of a major tree plantation pest?. Annals of Forest Science.

[ref-51] Santadino M, Lucia A, Duhour A, Riquelme M, Naspi C, Masuh H, Liljesthrom G, Coviella C (2017). Feeding preference of *Thaumastocoris peregrinus* on several *Eucalyptus* species and the relationship with the profile of terpenes in their essential oils. Phytoparasitica.

[ref-52] Santolamazza-Carbone S, Rodríguez-Illamamola A, Cordero Rivera A (2006). Thermal requirements and phenology of the *Eucalyptus* snout beetle *Gonipterus scutellatus* Gyllenhal. Journal of Applied Entomology.

[ref-53] Savage LJ (1972). Foundations of statistics.

[ref-54] Scanavaca Junior L, Garcia JN (2021). Eucalyptus subgenus Symphyomyrtus: sections: Exsertaria, Latoangulatae and Maidenaria. Scientia Agricola.

[ref-55] Schröder ML, Nahrung HF, de Souza NM, Lawson SA, Slippers B, Wingfield MJ, Hurley BP (2021). Distribution of *Gonipterus* species and their egg parasitoids in Australia: implications for biological control. Forests.

[ref-56] Schröder ML, Slippers B, Wingfield MJ, Hurley BP (2020). Invasion history and management of Eucalyptus snout beetles in the *Gonipterus scutellatus* species complex. Journal of Pest Science.

[ref-57] Souza NM, Junqueira LR, Wilcken CF, Soliman EP, Camargo MB, Nickele MA, Barbosa LR (2016). Ressurgência de uma antiga ameaça: Gorgulho-do-eucalipto *Gonipterus platensis* (Coleoptera: Curculionidae). https://www.ipef.br/publicacoes/ctecnica/nr209.pdf.

[ref-58] Statistical Analysis System (SAS^®^) (2001). User’s Guide: Statistics, Version 8.2.

[ref-59] Steinbauer MJ, Wanjura WJ (2002). Christmas beetles (*Anoplognathus* spp., Coleoptera: Scarabaeidae) mistake peppercorn trees for eucalypts. Journal of Natural History.

[ref-60] Tomé M, Almeida MH, Barreiro S, Branco MR, Deus E, Pinto G, Silva JS, Soares P, Rodríguez-Soalleiro RZ (2021). Opportunities and challenges of *Eucalyptus* plantations in Europe: the Iberian Peninsula experience. European Journal of Forest Research.

[ref-61] Tooke FGC (1955). The eucalyptus snout beetle, Gonipterus scutellatus Gyll, a study of its ecology and control by biological means.

[ref-62] Tukey J (1949). Comparing individual means in the analysis of variance. Biometrics.

[ref-63] Uesugi A (2015). The slow-growth high-mortality hypothesis: direct experimental support in a leafmining fly. Ecological Entomology.

[ref-64] Valente C, Afonso C, Gonçalves CI, Branco M (2019). Assessing the competitive interactions between two egg parasitoids of the Eucalyptus snout beetle, *Gonipterus platensis*, and their implications for biological control. Biological Control.

[ref-65] Valente C, Gonçalves CI, Monteiro F, Gaspar J, Silva M, Sottomayor M, Paiva MR, Branco M (2018). Economic outcome of classical biological control: a case study on the *Eucalyptus* snout beetle, *Gonipterus platensis*, and the parasitoid *Anaphes nitens*. Ecological Economics.

[ref-66] Valente C, Gonçalves CI, Reis A, Branco M (2017). Pre-selection and biological potential of the egg parasitoid *Anaphes inexpectatus* for the control of the Eucalyptus snout beetle, *Gonipterus platensis*. Journal of Pest Science.

[ref-67] Wilcken CF, de Oliveira NC, Sartório RC, Loureiro EB, BezerraJúnior N, Rosado Neto GH (2008). *Gonipterus scutellatus* Gyllenhal (Coleoptera: Curculionidae) occurrence in eucalyptus plantations in Espírito Santo State, Brazil. Arquivos do Instituto Biologico.

